# Cytoplasmic membrane vesicles from *Clostridioides difficile* R20291 are remodeled by osmotic stress

**DOI:** 10.3389/fmicb.2026.1868783

**Published:** 2026-06-17

**Authors:** Camila Queraltó, José Rodríguez, Fernando Escobar, Osvaldo Inostroza, Gustavo Saavedra, Giuliana Torres, Ruth González, Daniel Paredes-Sabja, Pablo Castro-Córdova, Alejadro Regaldiz, Katina Schinnerling, Jorge A. Soto, Iván L. Calderón, Juan A. Fuentes, Fernando Gil

**Affiliations:** 1 Departamento de Ciencias Biológicas, Facultad de Ciencias de la Vida, Universidad Andrés Bello, Santiago, Chile; 2 Microbiota-Host Interactions and Clostridia Research Group, Program of Microbiota and Applied Molecular Biology, Center for Biomedical Research and Innovation (CIIB), Universidad de los Andes, Santiago, Chile; 3 Programa de Doctorado en Biomedicina, Facultad de Medicina, Universidad de los Andes, Santiago, Chile; 4 Department of Biology, Texas A&M University, College Station, TX, United States; 5 IMPACT Center of Interventional Medicine for Precision and Advanced Cellular Therapy, Santiago, Chile; 6 Laboratory of Nano-Regenerative Medicine, Centro de Investigación e Innovación Biomédica (CiiB), Faculty of Medicine, Universidad de los Andes, Santiago, Chile; 7 Programa de Doctorado en Biociencias Moleculares, Facultad de Ciencias de la Vida, Universidad Andrés Bello, Santiago, Chile; 8 Centro de Investigación de Resiliencia a Pandemias, Facultad de Ciencias de la Vida e Instituto de Salud Pública, Universidad Andrés Bello, Santiago, Chile; 9 Laboratorio de Inmunología Traslacional, Facultad de Ciencias de la Vida, Universidad Andrés Bello, Santiago, Chile; 10 Millennium Institute on Immunology and Immunotherapy, Laboratorio de Inmunología TrasLacional, Centro de Investigación de Resiliencia a PanDemias, Facultad de Ciencias de la Vida, Universidad Andrés Bello, Santiago, Chile; 11 Laboratorio de RNAs Bacterianos, Facultad de Ciencias de la Vida, Universidad Andrés Bello, Santiago, Chile; 12 Laboratorio de Genética y Patogénesis Bacteriana, Facultad de Ciencias de la Vida, Universidad Andrés Bello, Santiago, Chile; 13 School of Medicine, Faculty of Medicine, Universidad de los Andes, Santiago, Chile

**Keywords:** *Clostridioides difficile*, cytoplasmic membrane vesicles, cytotoxicity, epithelial cells, NaCl

## Abstract

**Introduction:**

Bacterial extracellular vesicles are important mediators of host–microbe interactions, facilitating the transport of proteins, toxins, and other bioactive molecules across biological barriers. Although vesicle production has been reported in several Gram-positive pathogens, the environmental factors influencing vesicle characteristics in *Clostridioides difficile* remain poorly understood. This study investigated the impact of osmotic stress on the physicochemical properties and epithelial cell association of cytoplasmic membrane vesicles (CMVs) produced by the hypervirulent strain R20291.

**Methods:**

*C. difficile* R20291 was cultured under standard conditions or in medium supplemented with 350 mM NaCl. CMVs were isolated from culture supernatants by ultrafiltration and ultracentrifugation. Vesicle morphology, size distribution, and abundance were evaluated using transmission electron microscopy and nanoparticle tracking analysis. Protein and lipid contents were quantified using biochemical assays. CMV association with HT-29 epithelial cells was assessed by confocal microscopy and flow cytometry. Glucosyltransferase-associated enzymatic activity was evaluated using a colorimetric substrate assay before and after vesicle disruption.

**Results:**

CMVs were produced under both culture conditions and displayed the typical spherical morphology observed for bacterial membrane vesicles. Osmotic stress was associated with significant changes in vesicle physicochemical properties. NaCl-derived CMVs exhibited smaller particle diameters and a narrower size distribution than control CMVs. Biochemical analyses showed increased protein content and reduced lipid levels in vesicles produced under high-salinity conditions. Confocal microscopy and flow cytometry demonstrated increased CMV-associated fluorescence in HT-29 cells exposed to NaCl-derived vesicles compared with control vesicles. Vesicle preparations displayed measurable glucosyltransferase-associated enzymatic activity, which increased following vesicle disruption, suggesting the presence of enzyme-associated cargo within the vesicle structure.

**Discussion:**

These findings demonstrate that osmotic stress is associated with alterations in the size, composition, and epithelial cell association of CMVs produced by *C. difficile*. The observed changes suggest that environmental conditions may influence vesicle-mediated interactions between *C. difficile* and host cells, potentially affecting processes relevant to bacterial colonization and pathogenesis.

## Introduction

*Clostridioides difficile* is a leading cause of antibiotic-associated diarrhea and one of the most important pathogens responsible for healthcare-associated infections worldwide. Clinical manifestations of *C. difficile* infection (CDI) range from mild diarrhea to severe colitis, toxic megacolon, and life-threatening complications. Disease pathogenesis is primarily driven by the large glycosylating toxins TcdA and TcdB, which disrupt epithelial barrier integrity and trigger robust inflammatory responses in the intestinal mucosa (Smits et al., 2016; Aktories et al., 2017). However, toxin activity alone does not fully explain disease progression, which is also shaped by spore formation, host immune responses, microbiota composition, and environmental conditions within the gut (Theriot and Young, 2015; Kordus et al., 2022). In this context, bacterial extracellular vesicles (BEVs) also known as membrane vesicles (MVs) have emerged as key mediators of host–microbe interactions. BEVs are nanoscale lipid bilayer structures (20–500 nm) released by both Gram-negative bacteria, as outer membrane vesicles (OMVs), and Gram-positive organisms, as cytoplasmic membrane vesicles (CMVs) (Brown et al., 2015; Toyofuku et al., 2019). These vesicles encapsulate proteins, lipids, nucleic acids, and metabolites derived from the bacterial cytoplasm and membrane, enabling the protected delivery of biologically active cargo to target cells (Schwechheimer and Kuehn, 2015; Gill et al., 2019). In pathogenic bacteria, BEVs have been shown to transport toxins, enzymes, adhesins, and immune-modulatory molecules, thereby contributing to virulence and host-pathogen communication (Kaparakis-Liaskos and Ferrero, 2015; Toyofuku et al., 2019).

Importantly, BEV production and composition are highly dynamic and responsive to environmental conditions. Factors such as nutrient availability, growth phase, bile acids, and physicochemical stressors can reshape vesicle biogenesis, cargo selection, and functional properties (Toyofuku et al., 2019; Sartorio et al., 2023). Within the intestinal environment, bacteria are exposed to fluctuating osmolarity, pH, and metabolite concentrations, all of which can influence bacterial physiology and potentially modulate vesicle-mediated interactions (Theriot and Young, 2015; Kordus et al., 2022). In *C. difficile*, emerging evidence indicates that vesicles represent an additional layer of host interaction. Vesicles produced by toxigenic strains contain surface-associated proteins such as SlpA and can induce pro-inflammatory cytokine responses in epithelial cells, even in the absence of detectable free toxins (Nicholas et al., 2017; Fan et al., 2026). More recent studies suggest that *C. difficile* vesicles can modulate host metabolic and immune pathways and, notably, can disseminate systemically, reaching distal sites such as the placenta where they affect trophoblast function (Caballano-Infantes et al., 2023; Zha et al., 2024). These findings support a model in which vesicles act as multifunctional delivery systems contributing to disease beyond canonical toxin activity. Despite these advances, the environmental determinants regulating CMV production and function in *C. difficile* remain poorly defined. This represents a critical knowledge gap, given that virulence factor expression in this pathogen is tightly linked to environmental cues. High-salinity conditions, for instance, inhibit bacterial growth while promoting toxin gene expression, indicating that osmotic stress can directly modulate virulence-associated pathways (Michel et al., 2022). At the cellular level, hyperosmotic adaptation in *C. difficile* involves major remodeling of envelope physiology, including compatible solute accumulation and c-di-AMP–dependent regulation of potassium homeostasis, cell wall integrity, and osmotolerance (Michel et al., 2022; Oberkampf et al., 2022). Studies in other bacterial systems provide mechanistic support for a link between envelope stress and vesicle biogenesis. Perturbations in membrane or cell wall homeostasis can alter vesicle production, size distribution, and cargo composition. For example, bile salts modulate OMV production and virulence traits in Campylobacter jejuni, while cell wall imbalance enhances CMV release and enriches vesicles in virulence factors in *Staphylococcus aureus* (Elmi et al., 2017; Taheri et al., 2018; Davies et al., 2019; Li et al., 2024). Furthermore, vesicle biophysical properties such as size heterogeneity have been shown to influence epithelial uptake pathways and functional outcomes, as demonstrated for *Helicobacter pylori* OMVs (Turner et al., 2018). Collectively, these observations suggest that environmental stressors capable of remodeling the bacterial envelope may directly influence vesicle architecture and biological activity. Based on this framework, we hypothesized that hyperosmotic conditions reshape CMV biogenesis in *C. difficile*, thereby altering vesicle physicochemical properties and their association with host cells. To test this, we analyzed CMVs produced by the hypervirulent strain R20291 under control conditions and in the presence of NaCl. Vesicles were characterized using transmission electron microscopy, nanoparticle tracking analysis, and biochemical assays. In parallel, vesicle association with epithelial cells was assessed by confocal microscopy and flow cytometry, and toxin-associated enzymatic activity was evaluated using a glucosyltransferase assay. Our results demonstrate that osmotic stress significantly alters CMV size distribution, composition, and epithelial cell association, supporting a model in which environmental conditions modulate vesicle-mediated host–pathogen interactions in *C. difficile*.

## Materials and methods

### Bacterial strain and culture conditions

The hypervirulent *C. difficile* strain R20291 (PCR ribotype 027) was used throughout this study as a model organism to investigate the influence of osmotic stress on cytoplasmic membrane vesicle (CMV) production. This strain was kindly gifted by Daniel Paredes-Sabja and has been widely employed in studies addressing virulence regulation, host interaction and pathogenesis of *C. diffTicile* infection (Castro-Córdova et al., 2020; Álvarez et al., 2020; Queraltó et al., 2023; Inostroza et al., 2024; Queraltó et al., 2025). Bacterial cultures were routinely grown in brain heart infusion supplemented medium (BHIS; BD Difco, San Jose, CA, United States) containing 0.2% (w/v) sodium taurocholate, 0.1% (w/v) fructose and 0.1% (w/v) glucose. Taurocholate is commonly used to promote efficient germination and vegetative growth of *C. difficile*. Cultures were incubated at 37 °C under strictly anaerobic conditions in a BACTRON EZ anaerobic chamber (Shellab, Cornelius, OR, United States) containing an atmosphere of 5% hydrogen, 5% carbon dioxide and 90% nitrogen. To evaluate the effect of osmotic stress on vesicle production, bacterial cultures were grown under two experimental conditions: standard BHIS medium (control condition) and BHIS supplemented with 350 mM NaCl (NaCl condition). This concentration was selected based on previous physiological studies showing that increased salinity induces envelope stress responses in anaerobic bacteria while maintaining bacterial viability (Michel et al., 2022; Oberkampf et al., 2022). Starter cultures were initiated from freshly isolated colonies grown on BHIS agar plates and incubated overnight. Experimental cultures (250 mL) were inoculated at an initial optical density of OD_600_ ≈ 0.05 and grown for approximately 16 h until early stationary phase (OD_600_ ≈ 0.9), a growth stage frequently associated with increased vesicle production in bacterial systems. To ensure comparability between experimental conditions and to account for potential differences in bacterial growth, cultures were adjusted to equivalent CFU mL^–1^ prior to vesicle isolation.

### Isolation and purification of cytoplasmic membrane vesicles

Cytoplasmic membrane vesicles were isolated from bacterial culture supernatants using differential centrifugation and ultracentrifugation procedures adapted from established extracellular vesicle purification protocols widely used in studies of Gram-positive bacteria. Briefly, bacterial cultures grown under control or NaCl conditions were centrifuged at 5,400 × *g* for 30 min at 4 °C to remove intact bacterial cells. The resulting supernatants were carefully transferred to sterile tubes to avoid disturbing the pellet and subsequently filtered through 0.45 μm pore-size membrane filters (Millipore) to eliminate remaining cells and large debris. Filtered supernatants were concentrated using ultrafiltration devices with a 100 KDa molecular weight cutoff (Ultracel, Merck, Darmstadt, Germany), allowing enrichment of vesicle particles while removing soluble proteins and small molecules. Concentrated samples were subjected to ultracentrifugation at 150,000 × *g* for 3 h at 4 °C using a Beckman ultracentrifuge. Following ultracentrifugation, the vesicle-containing pellet was gently resuspended in Dulbecco’s phosphate-buffered saline (DPBS; Gibco, Waltham, MA, United States). Vesicle suspensions were aliquoted and stored at −80 °C until further use. This isolation approach has been widely applied in studies of bacterial extracellular vesicles and allows efficient recovery of vesicles secreted during bacterial growth (Nicholas et al., 2017; Briaud and Carroll, 2020, Fuentes et al., 2025).

### Transmission electron microscopy

Morphological characterization of vesicle suspensions was performed using transmission electron microscopy (TEM). Vesicle suspensions were deposited onto formvar/carbon-coated copper grids (Electron Microscopy Sciences, Hatfield, PA, United States) and incubated for approximately 1 min to allow adsorption of vesicles onto the grid surface. Excess liquid was removed using filter paper and grids were negatively stained using 3% uranyl acetate for one minute. Stained grids were examined using a Talos F200X G2 transmission electron microscope (Thermo Fisher Scientific). Negative-staining TEM is widely used to visualize bacterial extracellular vesicles and allows confirmation of vesicle morphology, membrane structure and particle integrity. Approximate sizes were performed using ImageJ software, counting 30 vesicles per condition as an approximate estimate. Previous studies of vesicles released by *C. difficile* have reported spherical vesicles with diameters typically ranging between 20 and 300 nm, consistent with extracellular vesicles described in other bacterial species. Because negative staining may introduce artifacts associated with dehydration or staining procedures, TEM observations were complemented with nanoparticle tracking analysis to obtain quantitative measurements of vesicle size distribution.

### Nanoparticle tracking analysis

Particle size distribution and concentration of vesicle suspensions were determined using nanoparticle tracking analysis (NTA) with a NanoSight NS300 instrument (Malvern Panalytical, United Kingdom). NTA is widely used for quantitative characterization of extracellular vesicles because it measures the Brownian motion of individual particles and calculates particle diameter using the Stokes–Einstein equation. Vesicle samples were diluted in sterile filtered DPBS to achieve particle concentrations appropriate for analysis (typically 20–100 particles per frame). Three independent videos were recorded for each sample under identical acquisition parameters and analyzed using NanoSight software to determine mean particle diameter, modal particle diameter, particle concentration and percentile values (D10, D50, and D90). To ensure accurate comparison between vesicle preparations obtained under different growth conditions, vesicle measurements were normalized according to particle concentration determined by nanoparticle tracking analysis (NTA). Vesicle suspensions were first quantified using a NanoSight NS300 instrument (Malvern Analytical), which tracks the Brownian motion of individual particles and calculates particle concentration and size distribution using the Stokes–Einstein equation. Vesicle samples used for downstream biochemical and cellular assays were normalized based on particle number, allowing equivalent particle input across experimental conditions. This normalization strategy minimizes biases associated with differences in vesicle yield between samples and is increasingly recommended for extracellular vesicle studies in both microbial and eukaryotic systems. Particle concentrations were expressed as particles per milliliter and vesicle preparations used in comparative assays were adjusted to identical particle concentrations prior to analysis. NTA-based normalization has become a standard approach for quantitative vesicle studies because it allows direct comparison of vesicle populations independent of variations in protein concentration or culture density (Dragovic et al., 2011; Gardiner et al., 2016). Vesicles were initially quantified and normalized according to particle concentration determined by NTA to ensure comparable vesicle recovery between conditions. For downstream epithelial cell association and stimulation assays, vesicle suspensions were subsequently adjusted to defined protein concentrations (50 or 100 μg/mL depending on the assay format) to ensure reproducible fluorescence detection and experimental consistency.

### Zeta potential measurements

The Zeta potential of CMVs was measured at room temperature (25 °C) using a Zetasizer Nano series MPT-Z multi-Purpose Titrator (Malvern, United Kingdom). The device was equipped with a Helium-Neon laser (633 nm), serving as a light source. Zetasizer measurements in aqueous media were conducted at a detection angle of 173.13°, with a measurement range covering diameters from 0.3 nm to 10 μm. Capillary cells DTS 1071 were employed for the measurements. CMVs were resuspended in ultrapure water for Zeta potential measurements. In this study, each measurement was conducted with a minimum of 50 events and up to a maximum of 100 events, contingent upon the quality of the results as determined by the Malvern Zetasizer software (version 7.11) calculations. Subsequently, 10 measurements were taken for each sample, resulting in a total range of 500–1,000 measurements per sample. Additionally, at least three independent biological replicates were performed for every measurement. The measurements provide insights into the surface charge characteristics of CMVs, as previously reported (Marchant et al., 2024), and the impact of NaCl on their Zeta potential.

### Quantification of vesicle protein and lipid content

Total protein content of vesicle preparations was quantified using a bicinchoninic acid (BCA) protein assay (Thermo Fisher Scientific) following the manufacturer’s instructions. Absorbance was measured at 562 nm using a microplate spectrophotometer, and protein concentrations were calculated using bovine serum albumin as a standard. This method is widely used for quantifying protein cargo in extracellular vesicle preparations and provides a robust estimate of vesicle-associated protein content. Total vesicle lipid content was determined using the membrane-intercalating fluorescent dye FM4-64 (Thermo Fisher Scientific). FM4-64 is a lipophilic styryl dye that becomes strongly fluorescent upon insertion into lipid bilayers and is commonly used to quantify membrane content in bacterial vesicle preparations. Vesicle samples were incubated with FM4-64 at 37 °C for approximately 10 min, allowing incorporation of the dye into vesicle membranes. Fluorescence intensity was then measured using a microplate reader with excitation at 485 nm and emission at approximately 670 nm. Relative lipid content was calculated from fluorescence values after subtraction of background fluorescence from dye-only controls. (Klimentová and Stulík, 2015; Brown et al., 2015). Proteins and lipids were normalized by CFU/ml as described by Nevermann et al. (2019).

### SDS–PAGE analysis of vesicle proteins

To evaluate differences in vesicle protein cargo, vesicle preparations corresponding to 50 μg total protein were mixed with Laemmli sample buffer and heated at 95 °C for 5 min. Samples were separated using 12% SDS-polyacrylamide gels following standard electrophoresis procedures. PageRuler Plus Prestained Protein Ladder (Thermoscientific) was used as molecular weight standard. After electrophoresis, gels were stained using One-Step Blue Protein Gel Stain (Biotium) to visualize protein band patterns. Differences in band distribution and intensity between vesicle preparations were evaluated qualitatively to identify potential variations in vesicle protein composition.

### Fluorescent labeling of vesicles and epithelial cell association assays

Vesicles were fluorescently labeled using the lipophilic membrane dye DiO (3,3’-dioctadecyloxacarbocyanine perchlorate; Thermo Fisher Scientific), which incorporates into lipid bilayers and enables visualization of vesicle membranes. Vesicle suspensions were incubated with 1% (v/v) DiO for 20 min at 37°C under gentle agitation to allow efficient dye incorporation into vesicle membranes. Following labeling, excess unbound dye was removed by repeated washing using centrifugal filtration devices with a 100 KDa molecular weight cutoff (Amicon Ultra, Millipore), ensuring removal of free dye molecules while retaining vesicle particles. A dye-only control consisting of phosphate-buffered saline subjected to the same labeling and filtration procedure was processed in parallel to control for background fluorescence and to verify the absence of dye aggregates. In addition, unlabeled vesicle preparations were included as negative controls to determine background fluorescence associated with vesicle preparations and host cells. For association experiments, HT-29 human colorectal epithelial cells were maintained in Dulbecco’s Modified Eagle Medium (DMEM; Gibco) supplemented with 10% fetal bovine serum and antibiotics at 37 °C in a humidified atmosphere containing 5% CO_2_. Cells were seeded onto sterile glass coverslips and allowed to reach approximately 70%–80% confluence prior to vesicle exposure. DiO-labeled vesicles were added to epithelial cell cultures and incubated for defined time periods under standard culture conditions. After incubation, cells were washed extensively with phosphate-buffered saline to remove unbound vesicles and subsequently fixed with 4% paraformaldehyde prior to imaging. Vesicle association with epithelial cells was first visualized using confocal laser scanning microscopy. HT-29 cells were cultured at a density of 500.000 cells per well in plates containing previously inserted 12 mm sterile coverslips. DiO-labeled CMVs (50 μg/mL) were added for 3 h at 37 °C (control 4 °C). After stimulation, the medium was removed, and cells were washed three times with PBS, fixed with 4% PFA for 20 min, and washed another three times with PBS. Cells on coverslips were counterstained with membrane dye WGA (2.5 μg/mL) and Hoechst 33,342 (2 μg/mL) nuclear stain for 20 min. Cells were washed three more times with PBS, and coverslips were mounted on Fluoromount-G mounting medium (ThermoFisher) on glass slides. Fluorescence images were obtained either on a Leica Sp8 confocal microscope (Wetzlar, Germany) at ×630 magnification (Sepúlveda-Pontigo et al., 2025). To obtain quantitative measurements, CMVs association was further analyzed using flow cytometry. To evaluate the kinetics of vesicle–cell association, HT-29 cells were stimulated with either CMVs (100 μg/mL) for 0, 3, 6, and 24 h. Different CMV concentrations were used because flow cytometry assays required a stronger fluorescent signal to reliably detect CMV-associated fluorescence across large cell populations, whereas confocal microscopy allowed visualization at lower concentrations. Cells were washed with PBS, detached with 10 mM EDTA in PBS for 20 min, and resuspended in PBS 0.5% BSA 1 mM EDTA (flow cytometry buffer) for acquisition with a BD FACSymphony A1 flow cytometer (BD Biosciences, Franklin Lakes, NJ, United states). Trypan blue was added to the samples at a ratio of 1:1 to eliminate extracellular fluorescence (quenching) due to attached CMVs. Control samples included untreated cells, cells incubated with dye-only preparations, and cells exposed to unlabeled vesicles to determine background fluorescence and establish gating parameters. Vesicle preparations used in these assays were normalized according to particle concentration determined by nanoparticle tracking analysis to ensure equivalent vesicle input across experimental conditions (Tashiro et al., 2017; Nicholas et al., 2017; Sepúlveda-Pontigo et al., 2025). Flow cytometry analyses were performed using a sequential gating strategy including exclusion of debris, doublets, and non-viable cells prior to quantification of DiO-positive HT-29 cells.

### Cytotoxicity assays and enzymatic activity measurements

Cytotoxic effects of vesicles were evaluated using an MTT cell viability assay (Mosmann, 1983). HT-29 cells were seeded in 96-well plates and incubated with vesicle preparations for 24 h. Cell viability was determined by measuring the conversion of MTT to formazan by metabolically active cells. Absorbance values were normalized to untreated control cells to calculate relative viability, and cell death was expressed as 100−% viability. This approach provides an indirect estimate of cytotoxicity based on metabolic activity. In parallel, toxin-associated enzymatic activity was evaluated using a PNPG-based glucosyltransferase assay designed to detect activity associated with glycosylating toxins such as TcdA and TcdB. Reaction mixtures containing PNPG substrate were incubated with vesicle samples and absorbance was measured spectrophotometrically following incubation. Vesicle disruption by sonication was used to determine whether enzymatic activity was associated with vesicle-encapsulated components (Brown et al., 2015). To provide appropriate positive controls for cytotoxicity and enzymatic assays, purified *C. difficile* toxins and toxin-enriched culture supernatants were included in parallel experiments. Purified TcdA and TcdB (600 pM final concentration) were used as reference standards to validate the sensitivity and dynamic range of the glucosyltransferase assay and to provide a benchmark for toxin-associated enzymatic activity. In addition, toxin-rich culture supernatants were obtained from late stationary-phase *C. difficile* cultures grown in BHIS medium under toxin-inducing conditions. Bacterial cells were removed by centrifugation followed by filtration through 0.45 μm membranes to obtain sterile supernatants containing secreted toxins. The concentration of purified toxins and toxin-containing supernatants was determined using a bicinchoninic acid (BCA) protein assay. For enzymatic assays, purified toxins were diluted to defined concentrations prior to incubation with the PNPG substrate, allowing comparison of vesicle-associated activity with soluble toxin activity. Quantification and normalization of toxin-containing samples ensured that differences observed in enzymatic activity reflected vesicle-associated effects rather than variations in toxin concentration. Similar experimental approaches for toxin purification, toxin quantification and cytotoxicity analysis have been widely used in studies investigating toxin production and virulence mechanisms in *C. difficile* (Carter et al., 2015; Govind and Dupuy, 2012; Olling et al., 2012; Shen, 2017; Queraltó et al., 2023).

### Statistical analysis

All experiments were performed using at least three independent biological replicates. Quantitative data are presented as mean ± standard deviation (SD). Statistical analyses were performed using GraphPad Prism software. Comparisons between two experimental groups were conducted using unpaired two-tailed Student’s *t*-tests. For experiments involving multiple conditions, statistical significance was evaluated using one-way analysis of variance (ANOVA) followed by Tukey’s multiple comparison test. Differences were considered statistically significant when *p* < 0.05.

## Results

To investigate whether environmental salinity influences vesicle production in *C. difficile*, vesicles were isolated from cultures grown under standard laboratory conditions (CTRL) or under high-salinity stress (NaCl) and characterized using structural, biophysical and functional approaches (Welsh et al., 2024). These analyses revealed that osmotic stress is associated with measurable changes in vesicle size distribution, yield and biochemical composition.

### Morphological characterization of cytoplasmic membrane vesicles

**FIGURE 1:**
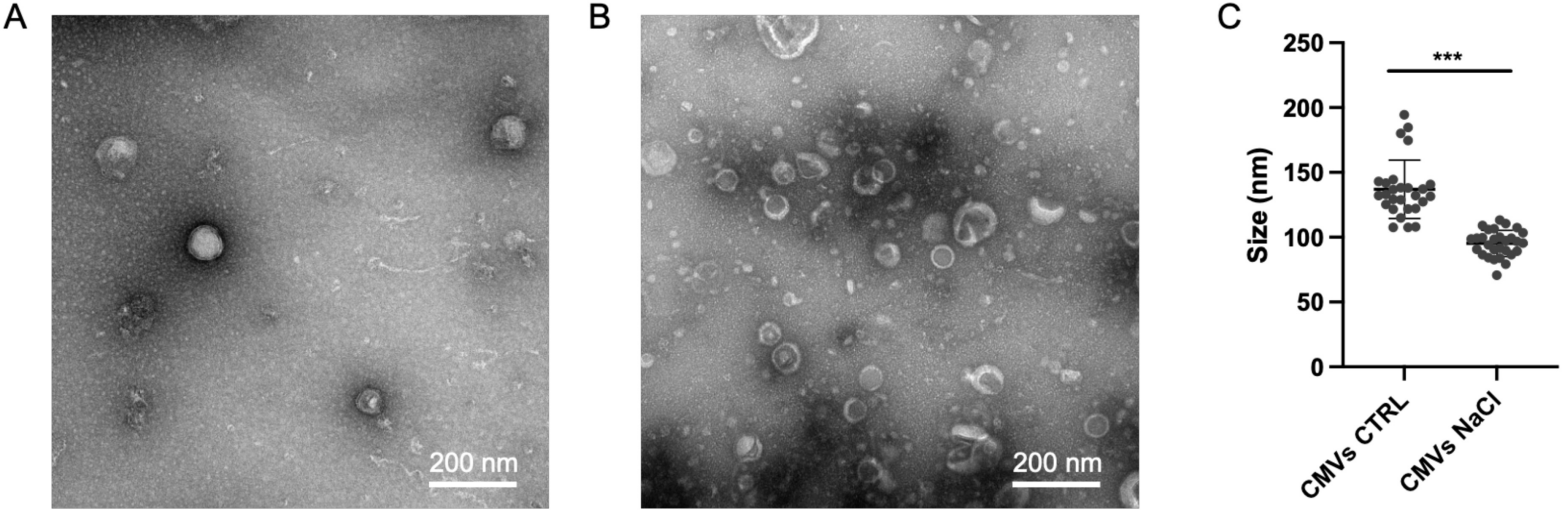
Morphological characterization of cytoplasmic membrane vesicles produced by *Clostridioides difficile*. Transmission electron microscopy (TEM) images of cytoplasmic membrane vesicles (CMVs) isolated from *Clostridioides difficile* R20291 cultures grown under control conditions [CTRL, **(A)]** or in medium supplemented with 350 mM NaCl **(B)**. Scale bars indicate the magnification used for vesicle visualization. Representative images of *n* = 6. **(C)** Quantification of CMVs size distribution from TEM images (*n* = 30).

To determine whether *C. difficile* produces cytoplasmic membrane vesicles (CMVs) under the experimental conditions used in this study, vesicle preparations obtained from cultures grown under CTRL or in medium supplemented with 350 mM NaCl were examined using transmission electron microscopy (TEM). Representative micrographs are shown in Figures 1A, B. TEM analysis confirmed the presence of spherical membrane-delimited vesicle-like structures in both experimental conditions. These structures displayed diameters within the nanoscale range and exhibited the characteristic morphology previously described for extracellular vesicles produced by *C. difficile* (Nicholas et al., 2017; Caballano-Infantes et al., 2023). Figure 1C shows a plot with approximate vesicles size calculated using ImageJ from six photos of each condition, counting 30 vesicles per condition as an approximate estimate (CTRL: 136.97 ± 22.52 nm; NaCl: 95.23 ± 9.34 nm).

### High salinity alters vesicle size distribution and yield

**FIGURE 2:**
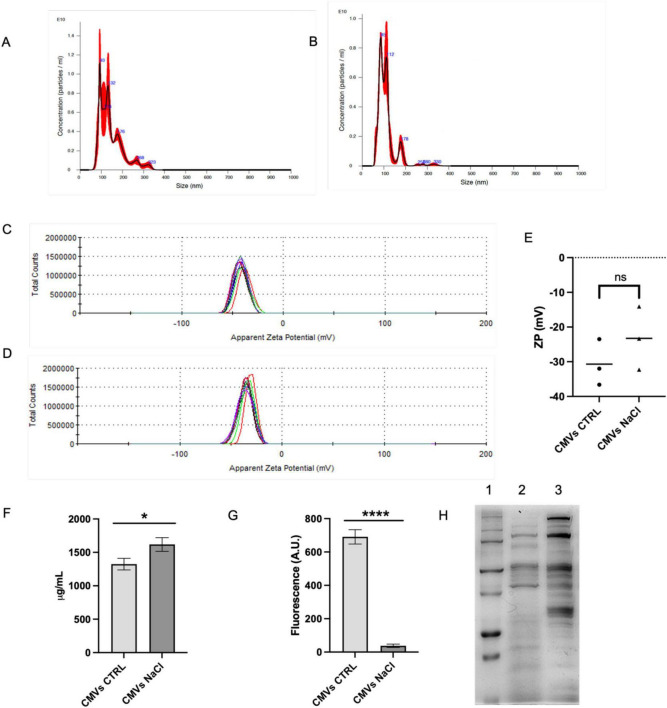
Physicochemical characterization of cytoplasmic membrane vesicles produced under control and high-salinity conditions. **(A,B)** Particle size distribution of vesicle populations determined by nanoparticle tracking analysis (NTA). **(C,D)** Zeta potential measurements of vesicle preparations. **(E)** Graphical summary of zeta potential values. **(F)** Quantification of total vesicle protein content (BCA assay). **(G)** Total lipid content determined using FM4-64 probe. **(H)** SDS–PAGE analysis of vesicle protein profiles showing differences in band intensity and distribution between conditions. (1): MW standard (PageRuler Plus Prestained Protein Ladder, Thermoscientific); (2): CTRL CMVs proteins; (3): NaCl CMVs proteins. Quantitative data represent mean ± standard deviation (SD) from three independent biological replicates (*n* = 3). Statistical comparisons between groups were performed using unpaired two-tailed Student’s *t*-test. Statistical significance was defined as *p < 0.05, ****p < 0.0001.

Quantitative characterization of vesicle populations was performed using nanoparticle tracking analysis (NTA), and the resulting particle size distributions are shown in Figures 2A,B and Supplementary Table 1. Analysis of vesicle size distributions revealed consistent differences between vesicles produced under standard growth conditions and those generated in the presence of elevated NaCl concentrations. CMVs derived from NaCl-grown cultures displayed smaller particle diameters across multiple NTA metrics. In particular, the mean vesicle diameter decreased from 145.6 ± 1.2 nm in control CMVs to 10^8^.7 ± 3.4 nm in NaCl-derived CMVs, indicating a reduction in average vesicle size under osmotic stress conditions. A similar trend was observed for modal particle diameter, which shifted from 92.6 nm in control vesicles to 84.7 nm in NaCl vesicles, suggesting an enrichment of smaller vesicle populations in cultures exposed to increased salinity. Percentile analysis further supported this observation. The D10 value decreased from 90.1 to 75.1 nm, indicating an increased proportion of small vesicles in NaCl preparations. Likewise, the median particle size (D50) decreased from 130.8 to 100.1 nm, reflecting a shift in the central tendency of the vesicle population. The D90 value also decreased substantially, from 222.8 nm in control vesicles to 166.6 nm in NaCl-derived vesicles, suggesting a reduction in the larger vesicle population observed under control conditions. Consistent with these measurements, the standard deviation of particle size distributions was lower for NaCl-derived vesicles, indicating a narrower and less heterogeneous vesicle population. Particle concentration measurements indicated that vesicle yields differed between the two conditions. Control cultures produced vesicles at a concentration of 7.60 × 10^11^ particles mL^–1^, whereas NaCl-grown cultures yielded 4.39 × 10^11^ particles mL^–1^, representing an approximately 1.7-fold reduction in particle concentration. Importantly, cultures were normalized to equivalent CFU mL^–1^ prior to vesicle isolation to ensure comparability between conditions. Normalization of vesicle counts to bacterial load (1.5 × 10^8^ CFU/mL) indicated that control cultures produced approximately 5.07 × 10^3^ vesicles per CFU, whereas NaCl-grown cultures produced approximately 2.93 × 10^3^ vesicles per CFU, consistent with a reduction in vesicle production under high-salinity conditions. Moreover, the number of particles detected per frame and the number of tracked centers per frame were within the optimal detection range for nanoparticle tracking analysis in both experimental conditions, indicating that the measurements were obtained under comparable acquisition parameters. Together, the parameters summarized in Supplementary Table 1 indicate that elevated salinity is associated with a shift toward smaller vesicle diameters and reduced vesicle yield, while maintaining particle detection conditions within the optimal range for nanoparticle tracking analysis. These observations suggest that osmotic stress influences the size distribution and production of CMVs in *C. difficile*.

### Osmotic stress alters vesicle biochemical composition

To determine whether osmotic stress influences vesicle composition, vesicle preparations were further analyzed for surface charge, protein content and lipid content. Zeta potential measurements indicated that vesicles derived from both experimental conditions exhibited comparable negative surface charge values (Figures 2C–E), suggesting that electrostatic surface properties of vesicles were not substantially altered by salinity stress. Quantification of vesicle protein content using the bicinchoninic acid assay revealed that vesicles derived from NaCl-grown cultures exhibited increased relative protein content compared with vesicles produced under control conditions (Figure 2F). Normalization of vesicle counts to total protein content revealed that control-derived CMVs exhibited approximately 5.74 × 10^8^ particles per μg protein, whereas NaCl-derived vesicles showed approximately 2.71 × 10^8^ particles per μg protein, indicating a reduction in particle-to-protein ratio under high-salinity conditions (∼2.1-fold). In contrast, lipid quantification demonstrated reduced lipid content in vesicles derived from NaCl cultures relative to control vesicles (Figure 2G). These observations indicate that osmotic stress influences the relative abundance of protein and lipid components in vesicle preparations.

### Vesicle protein profiles differ between experimental conditions

To further examine potential differences in vesicle composition, protein profiles of vesicle preparations were analyzed using SDS–PAGE. The resulting electrophoretic patterns are shown in Figure 2H. Differences in CMV-associated protein banding patterns were observed between CTRL- and NaCl-derived vesicles, suggesting that osmotic stress may influence protein profiles detected in vesicle preparations. Several bands appeared more intense in NaCl-derived vesicles, whereas other bands detected in control vesicles were reduced or absent. These qualitative differences in electrophoretic profiles suggest that environmental salinity may influence vesicle protein cargo composition. These observations are qualitative and do not allow identification of specific vesicle-associated proteins.

### Association of CMVs with epithelial cells

**FIGURE 3:**
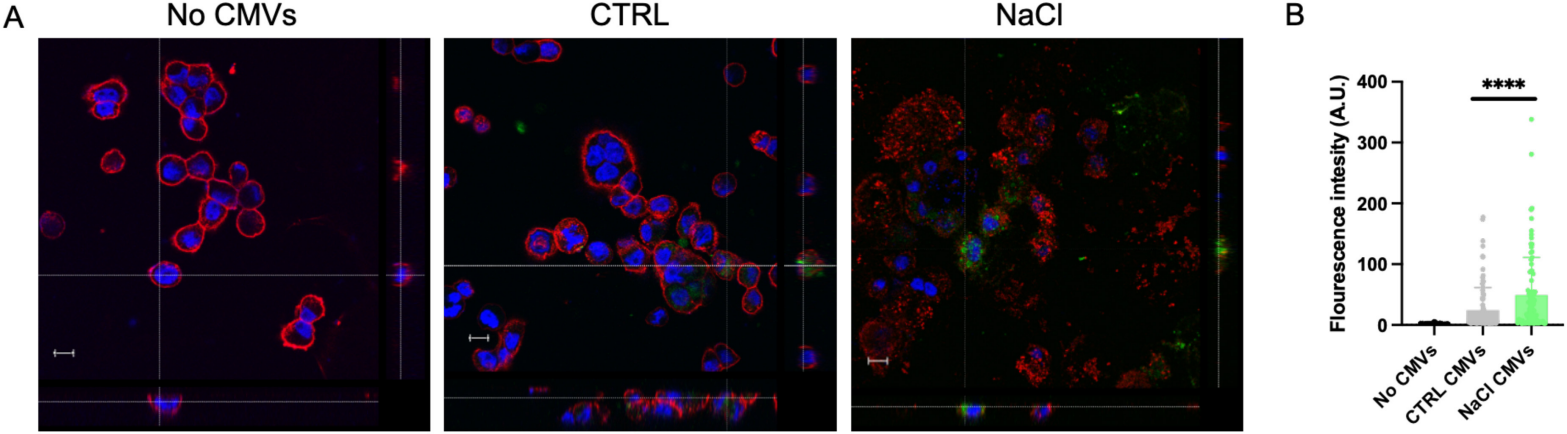
Confocal microscopy analysis of vesicle association with epithelial cells. HT-29 epithelial cells were incubated with DiO-labeled cytoplasmic membrane vesicles derived from *C. difficile* cultures grown under control conditions or in medium supplemented with 350 mM NaCl. **(A)** Representative confocal microscopy images show vesicle-associated fluorescence (green) corresponding to labeled vesicles interacting with epithelial cells. Cell nuclei were stained with Hoechst (blue) and membrane with WGA (red). Z-stack images confirm the presence of vesicle-associated fluorescence throughout cellular optical sections. Scale bars represent 10 μm. **(B)** Quantification of vesicle-associated fluorescence intensity from confocal microscopy images of HT-29 cells exposed to CMVs derived from control and NaCl conditions. Fluorescence intensity was quantified using region-of-interest (ROI) analysis from confocal images from individual cells and is presented as mean ± SD from independent biological replicates. Statistical comparisons between groups were performed using unpaired two-tailed Student’s *t*-test. Statistical significance was defined as ****p < 0.0001.

To investigate whether vesicles associate with intestinal epithelial cells, HT-29 cells were incubated with DiO-labeled vesicles derived from *C. difficile* cultures grown under control or NaCl conditions and analyzed using confocal microscopy. Representative images are shown in Figure 3A. Cells exposed to labeled vesicles displayed punctate fluorescence signals corresponding to vesicle-associated signal within or near epithelial cells. Cells incubated with vesicles derived from NaCl-grown cultures exhibited noticeably stronger fluorescence signals compared with cells exposed to control vesicles. Z-stack imaging further confirmed the presence of vesicle-associated fluorescence associated with cellular optical sections, supporting vesicle association with epithelial cells. Quantitative analysis of confocal fluorescence images further supported these observations. Cells exposed to CMVs derived from NaCl-grown cultures exhibited a significantly higher vesicle-associated fluorescence signal compared with cells treated with control vesicles, indicating increased association of NaCl-derived vesicles with epithelial cells (Figure 3B). These differences were consistently observed across independent experiments. Controls are showed in Supplementary Figure 1. These observations indicate that vesicles produced under high-salinity conditions exhibit increased CMV-associated fluorescence with epithelial cells compared with vesicles produced under standard growth conditions.

### Quantification of vesicle association using flow cytometry

**FIGURE 4:**
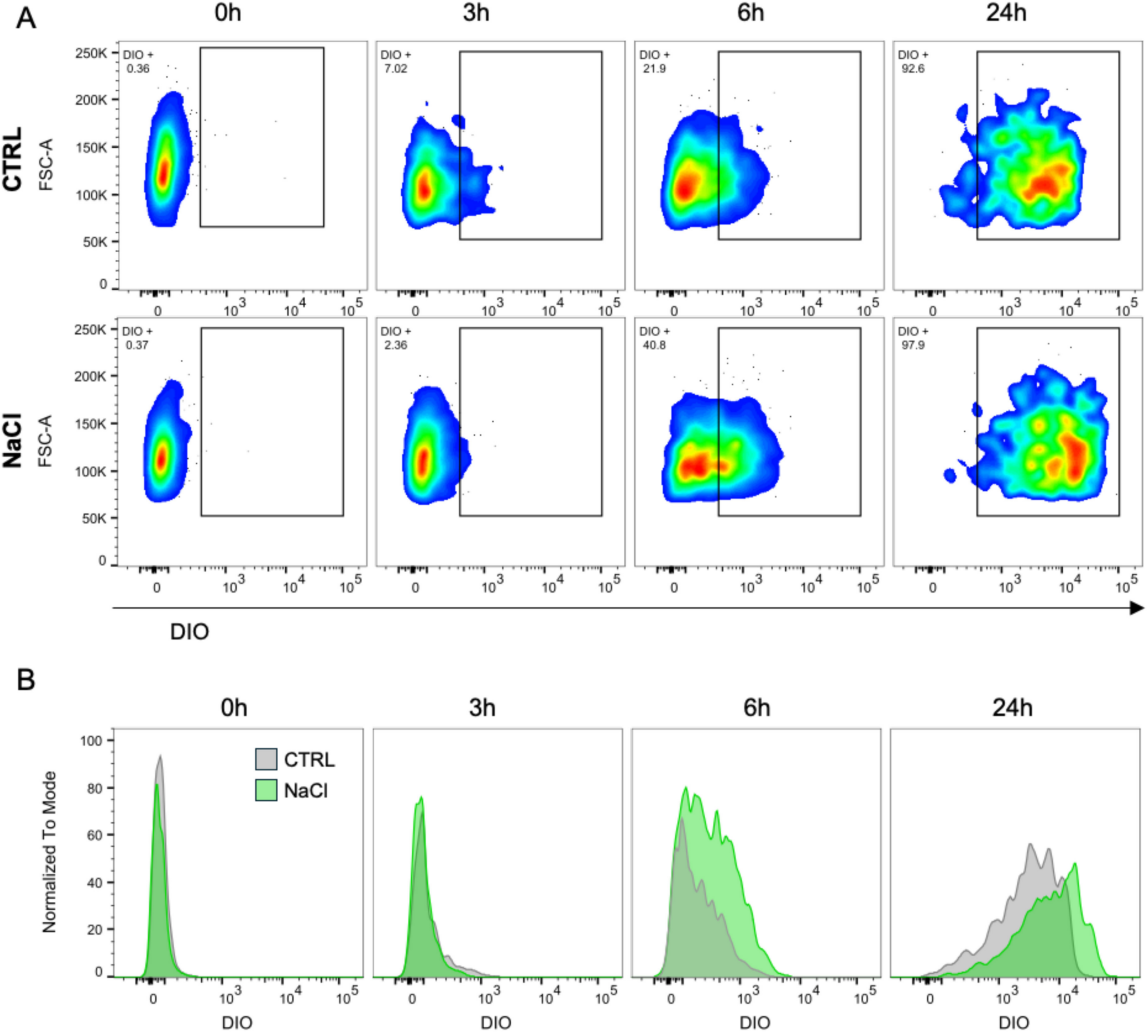
Flow cytometry quantification of vesicle association with epithelial cells. **(A)** HT-29 cells were incubated with DiO-labeled vesicles (0–24 h) derived from *C. difficile* cultures grown under CTRL or NaCl conditions and analyzed by flow cytometry using trypan blue as a quencher. **(B)** Histograms represent fluorescence intensity distributions corresponding to vesicle-associated signal in epithelial cells. Images shown are representative of three independent biological experiments.

To quantitatively assess vesicle association with epithelial cells, HT-29 cells incubated with DiO-labeled vesicles were analyzed using flow cytometry. Representative flow cytometry analysis revealed a progressive increase in DiO-positive events following exposure to CMVs (Figure 4A), indicating enhanced vesicle-associated fluorescence over time in both experimental conditions. Baseline fluorescence in untreated HT-29 cells remained minimal (<1% DiO-positive cells), confirming low background signal and negligible autofluorescence. In contrast, CMV-treated samples (NaCl condition) showed a marked and stepwise increase in DiO signal intensity and population spread. Early exposure resulted in a modest increase in DiO-positive cells in CTRL (2.36%) vs. NaCl (7.02%), while intermediate conditions led to a substantial shift in fluorescence distribution (CTRL: 21.9% – NaCl: 40.8%), suggesting increased vesicle–cell association. At the highest condition tested, nearly the entire population became DiO-positive (CTRL: 92.6 – NaCl: 97.9%), accompanied by a broad fluorescence distribution, indicative of heterogeneous but extensive vesicle association.

Density plots further supported these observations (Figure 4B), showing a clear rightward shift in fluorescence intensity for NaCl-derived CMVs compared to controls. NaCl-derived CMVs exhibited a broader fluorescence distribution and higher signal intensity compared with CTRL-derived CMVs, suggesting greater heterogeneity and increased association with epithelial cells. In contrast, CTRL-derived CMVs maintained a relatively narrow fluorescence distribution with lower overall intensity across condition. Collectively, these results demonstrate that CMVs produced under NaCl stress exhibit significantly increased CMV-associated fluorescence with target cells, as reflected by increased DiO labeling intensity and a higher proportion of positive events. Gating strategy and control without trypan blue are showed in Supplementary Figure 2. In Supplementary Figure 2A, at 24 h post-exposure, a substantial proportion of DiO-positive HT-29 cells displayed reduced viability, particularly in cells exposed to NaCl-derived CMVs, suggesting that increased CMV-associated fluorescence occurred in the context of compromised epithelial integrity. Importantly, quantification of DiO-positive cells was performed after exclusion of non-viable cells during flow cytometry gating, indicating that CMV-associated fluorescence was primarily detected in viable epithelial cells.

### Cytotoxic effects of vesicle preparations

**FIGURE 5:**
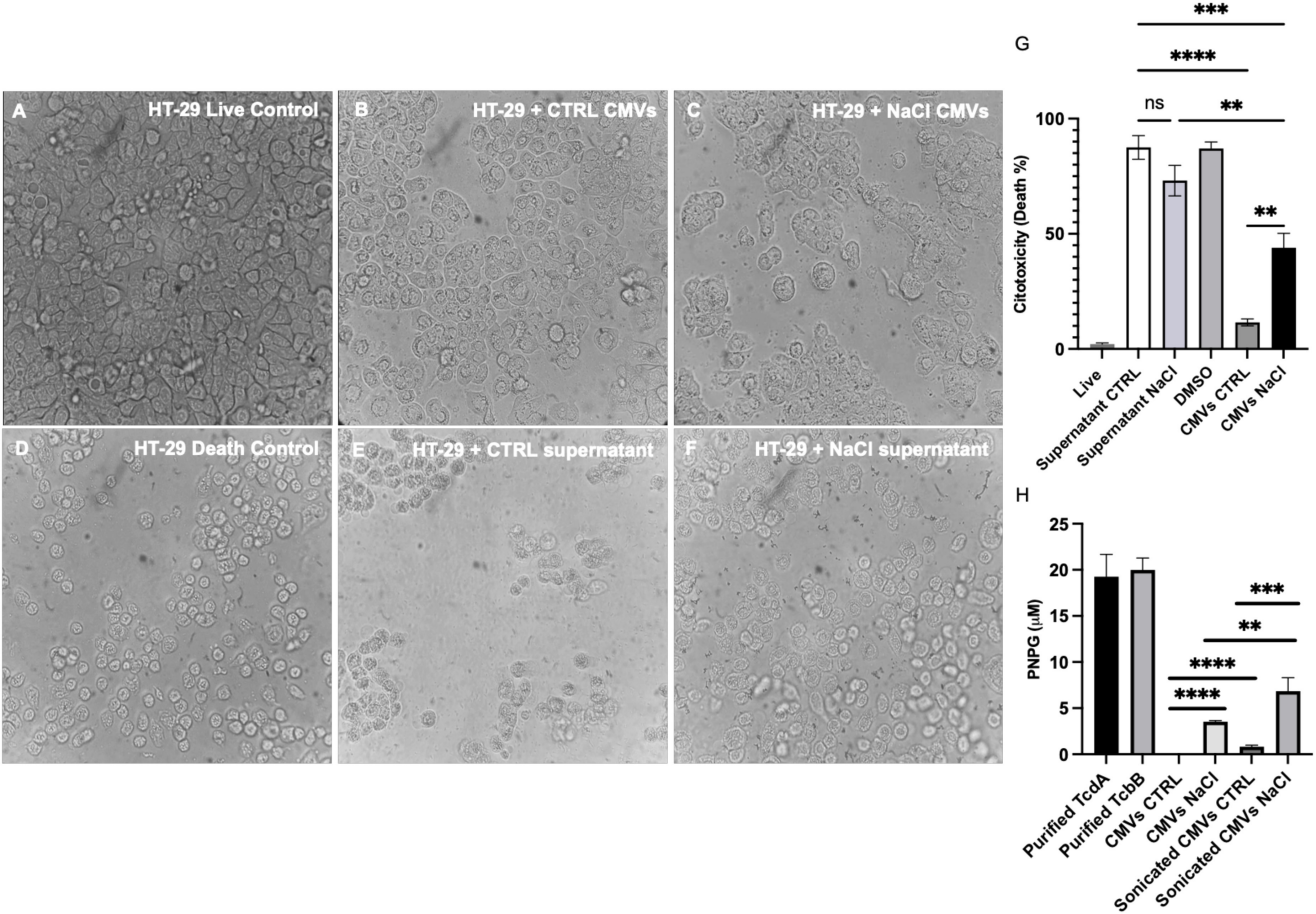
HT-29 cells were exposed to vesicles (100 μg/mL) derived from control or NaCl cultures for 24 h and cytotoxicity was assessed using an MTT assay. **(A)** Untreated HT-29 cells showing normal epithelial morphology. **(B)** HT-29 cells incubated with CMVs derived from control cultures (CMVs CTRL). **(C)** HT-29 cells incubated with CMVs derived from NaCl-treated cultures (CMVs NaCl). **(D)** Positive control of cell death (DMSO). **(E)** HT-29 cells treated with supernatant from control cultures (Supernatant CTRL). **(F)** HT-29 cells treated with supernatant from NaCl-exposed cultures (Supernatant NaCl). **(G)** Quantification of cytotoxicity assessed by MTT assay. Cell death percentage was calculated relative to untreated control cells. **(H)** Quantification of glucosyltransferase activity expressed as produced PNPG (μM). Data represent mean ± SD from three independent biological replicates (*n* = 3). Statistical analysis was performed using one-way ANOVA followed by Tukey’s multiple comparison test. Differences were considered statistically significant at **p < 0.01, ***p < 0.001, ****p < 0.0001.

Finally, to assess whether CMV–cell association were associated with functional effects on epithelial cell viability, the potential cytotoxic effects of vesicle preparations were evaluated using an MTT assay. The results of these experiments are shown in Figure 5. Exposure of HT-29 cells to vesicle preparations resulted in measurable reductions in cell viability relative to untreated controls. As expected, exposure to toxin-containing culture supernatants produced the strongest reduction in epithelial cell viability. Importantly, vesicles derived from NaCl-grown cultures produced greater reductions in epithelial cell viability compared with vesicles derived from control cultures. However, the magnitude of cytotoxicity observed for vesicle preparations remained lower than that produced by toxin-containing supernatants.

To determine whether cytoplasmic membrane vesicles (CMVs) contained toxin-associated enzymatic activity, vesicle preparations were analyzed using a PNPG-based glucosyltransferase assay. The results are summarized in Figure 5H. As expected, purified toxins used as positive controls displayed strong enzymatic activity, with TcdA and TcdB generating PNPG hydrolysis values of 19.25 and 19.98 μM, respectively. These values confirmed that the assay reliably detected glucosyltransferase activity associated with the major *C. difficile* toxins. In contrast, vesicle preparations derived from control cultures exhibited no detectable activity, suggesting that under standard growth conditions CMVs contained little or no accessible toxin-associated enzymatic activity. Interestingly, vesicles produced under high-salinity conditions displayed measurable glucosyltransferase activity, with NaCl-derived CMVs generating 3.53 μM PNPG hydrolysis, indicating the presence of enzymatic activity within these vesicle preparations. To evaluate whether vesicle integrity influenced enzymatic detection, vesicle samples were subjected to sonication prior to the assay. Sonication of control CMVs resulted in a small increase in detectable activity (0.83 μM PNPG), whereas disruption of NaCl-derived vesicles led to a marked increase in enzymatic activity (6.84 μM PNPG).

The increase in enzymatic activity observed following vesicle disruption is consistent with partial protection of enzymatic activity by membrane-associated structures and becomes more accessible upon vesicle lysis. These findings are consistent with membrane-associated enzymatic activity within CMV preparations. However, the contribution of vesicle-associated versus co-purified soluble components cannot be fully excluded under the current experimental conditions. Together, these results suggest that CMVs produced under high-salinity conditions contain detectable glucosyltransferase activity and that vesicle disruption enhances the accessibility of this activity in the assay.

## Discussion

Environmental conditions within the intestinal tract play a central role in shaping the physiology and virulence of *C. difficile*. Variations in nutrient availability, bile acid composition, osmolarity and microbial metabolites can influence bacterial metabolism, toxin expression and colonization dynamics (Smits et al., 2016; Kordus et al., 2022). In the present study, we investigated whether osmotic stress affects the production and biological properties of cytoplasmic membrane vesicles (CMVs) released by the hypervirulent strain R20291. Our results indicate that growth under high-salinity conditions is associated with measurable changes in vesicle size distribution, biochemical composition and epithelial cell association, suggesting that environmental osmotic conditions may influence vesicle-associated processes in *C. difficile*.

Transmission electron microscopy confirmed the presence of membrane-delimited vesicle-like structures in preparations obtained from cultures grown under both experimental conditions. The vesicles displayed a spherical morphology consistent with bacterial extracellular vesicles previously described in Gram-positive organisms (Brown et al., 2015; Toyofuku et al., 2019). Vesicle production has been reported in several Gram-positive pathogens including *S. aureus*, *Bacillus anthracis*, and *C. difficile*, where vesicles have been shown to transport proteins, toxins and other biologically active molecules capable of interacting with host cells (Rivera et al., 2010; Wang et al., 2018; Nicholas et al., 2017). In agreement with these reports, vesicles observed in our study exhibited diameters within the nanoscale range typical of bacterial extracellular vesicles.

Although TEM observations primarily provide qualitative information, quantitative NTA revealed a clear shift toward smaller vesicle populations under high-salinity conditions. Normalization of vesicle production to bacterial counts further indicated a reduction in vesicle output per cell under high-salinity conditions. Although these estimates are based on an approximate CFU value, they are consistent with a decrease in vesicle production per bacterium, suggesting that osmotic stress may be associated with changes in vesicle production and properties. Moreover, the reduction in mean and median vesicle diameter observed in NaCl-derived preparations suggests that osmotic stress may influence vesicle biogenesis or membrane remodeling processes. Vesicle size distributions are influenced by several factors including membrane curvature, envelope composition and cellular stress responses (Toyofuku et al., 2019, 2023). Environmental stresses that alter membrane tension or peptidoglycan structure have been shown to modify vesicle production and size distribution in multiple bacterial species. Therefore, the shift toward smaller vesicle populations observed in NaCl-grown cultures may reflect physiological adaptation to osmotic stress rather than a simple consequence of altered growth conditions.

In addition to differences in particle size, biochemical analyses indicated differences in vesicle composition between control and NaCl-derived vesicles. Vesicles produced under high salinity conditions displayed increased protein content together with reduced lipid levels relative to vesicles produced under standard laboratory conditions. Zeta potential measurements indicated no significant differences in surface charge between vesicles derived from control and NaCl-grown cultures, suggesting that global electrostatic properties of CMVs remain largely unchanged under osmotic stress. The increase in relative protein content together with the reduction in lipid-associated signal observed in NaCl-derived CMVs may reflect stress-associated remodeling of the bacterial envelope and vesicle biogenesis processes. Osmotic stress is known to alter membrane and cell wall physiology, which can influence vesicle size and composition (Toyofuku et al., 2019; Michel et al., 2022; Briaud and Carroll, 2020). The shift toward smaller vesicles observed under high-salinity conditions may contribute to a higher protein-to-lipid ratio, potentially due to geometric effects associated with reduced vesicle size as well as enrichment in surface-associated or stress-related proteins. In addition, environmental stress may favor the incorporation of proteins involved in envelope stability, host interaction or stress adaptation into vesicle cargo (Toyofuku et al., 2019; Michel et al., 2022; Sartorio et al., 2023). Since fluorescence analyses were normalized according to CMV-associated protein concentration rather than absolute particle number, differences in vesicle abundance and size distribution between conditions may have influenced fluorescence intensity measurements. Consequently, the increased lipid-associated fluorescence observed in CTRL-derived CMVs should be interpreted cautiously, as it may reflect combined effects of membrane composition and vesicle population characteristics. Although these interpretations remain speculative, they are consistent with the observed changes in particle-to-protein ratio and support the idea that osmotic stress reshapes vesicle composition rather than simply affecting vesicle abundance. Osmotic stress is known to induce global changes in bacterial physiology and protein expression. Therefore, the altered CMV-associated protein profiles observed under high salinity conditions may reflect both stress-induced remodeling of vesicle cargo and broader changes in cellular protein composition (Figure 2H). Because whole-cell and extracellular soluble protein fractions were not comparatively analyzed, the present study cannot definitively distinguish selective cargo enrichment from general alterations in bacterial protein expression under osmotic stress. Changes in vesicle composition in response to environmental signals have been reported in several bacterial species and may reflect adaptive changes in vesicle composition in response to environmental stress (Toyofuku et al., 2019; Sartorio et al., 2023). This observation suggests that the enhanced epithelial cell association observed for NaCl-derived vesicles is unlikely to be driven by differences in surface charge alone and instead may reflect changes in vesicle composition or surface-associated molecules. Consistent with this observation, normalization of particle counts to total protein content revealed a reduced particle-to-protein ratio under high-salinity conditions, indicating that vesicles produced during osmotic stress may contain relatively higher protein content per particle. This finding supports the notion that environmental conditions influence vesicle composition. For example, vesicles produced by intestinal bacteria may include proteins involved in host interaction and immune modulation, indicating that vesicle cargo is not merely a passive reflection of membrane composition but may instead represent a regulated process influenced by environmental conditions (Sartorio et al., 2023).

Vesicle association with epithelial cells represents another important functional aspect of bacterial extracellular vesicles. Confocal microscopy and flow cytometry analyses performed in this study demonstrated that CMVs derived from NaCl-grown cultures exhibited increased association with HT-29 epithelial cells compared with vesicles produced under control conditions. This observation was further supported by quantitative analysis of confocal fluorescence images, which revealed a significantly higher vesicle-associated signal in cells exposed to NaCl-derived CMVs compared with control vesicles (Figure 3B). Interaction of bacterial vesicles with host cells has been described for numerous bacterial species and can involve mechanisms such as membrane fusion, endocytosis or receptor-mediated uptake (Kaparakis-Liaskos and Ferrero, 2015). Vesicles produced by intestinal bacteria are capable of crossing mucus barriers and delivering bacterial molecules to host cells, thereby influencing immune signaling pathways and epithelial responses (Cañas et al., 2018; Durant et al., 2020). In the context of *C. difficile*, vesicles have previously been shown to interact with epithelial cells and induce pro-inflammatory responses even in the absence of detectable levels of classical toxins (Nicholas et al., 2017). Flow cytometry analyses performed in our study incorporated trypan blue quenching to minimize the contribution of extracellular fluorescence associated with vesicles bound to the cell surface. Under these conditions, a progressive increase in fluorescence intensity was observed (Figure 4A), supporting vesicle association that is resistant to extracellular quenching and therefore compatible with internalization or tight membrane interaction. In contrast, analysis performed in the absence of trypan blue (Supplementary Figure 2) showed a broader fluorescence signal with a higher proportion of DiO-positive events, consistent with the combined contribution of both surface-bound and internalized vesicles. The comparison between these conditions suggests that NaCl-derived CMVs exhibit increased cellular association that cannot be explained solely by surface adhesion, although the relative contribution of internalization versus strong membrane binding remains to be determined. The reduced structural integrity observed in HT-29 cells exposed to NaCl-derived CMVs suggests that increased CMV-associated fluorescence at later time points may occur in the context of compromised epithelial viability. Therefore, fluorescence signals detected under these conditions must be interpreted carefully, as they may reflect a combination of vesicle association with damaged cells, membrane disruption, and/or interaction with cellular debris rather than exclusively active vesicle internalization. Although reduced epithelial viability was observed at later time points following exposure to NaCl-derived CMVs, flow cytometry analyses were performed using a gating strategy that excluded non-viable cells prior to quantification of DiO-positive populations. Therefore, CMV-associated fluorescence was primarily detected in viable epithelial cells. Nevertheless, because osmotic stress-derived CMVs induced detectable cytotoxic effects and morphological alterations in HT-29 cells, fluorescence signals observed under these conditions remain to be fully confirmed, as they may reflect a combination of vesicle association with stressed cells, altered membrane integrity, and potential vesicle internalization rather than exclusively active uptake mechanisms. Although bacterial extracellular vesicles can be internalized through multiple pathways, including clathrin-mediated endocytosis and macropinocytosis in other systems, the specific mechanisms governing CMV uptake in *C. difficile* remain poorly defined and were not directly assessed in this study. Given the molecular weight of *C. difficile* toxins, their detection by SDS-PAGE would require targeted approaches, and therefore the observed band differences likely reflect broader compositional changes rather than specific toxin enrichment. Considering these observations, vesicles in our study induced measurable cytotoxic effects in epithelial cells. Because NaCl-derived CMVs induced detectable cytotoxic effects and reduced epithelial cell viability at later time points, conclusions regarding specific alterations in membrane-associated interactions must be interpreted carefully. Under these conditions, increased CMV-associated fluorescence may reflect multiple non-exclusive processes, including enhanced vesicle association with stressed epithelial cells, altered membrane integrity, or interactions with damaged cellular structures. Therefore, the present findings support increased CMV association with epithelial cells under osmotic stress conditions, but do not allow definitive conclusions regarding the underlying mechanisms of membrane interaction or active vesicle internalization. Although the magnitude of cytotoxicity was lower than that produced by toxin-containing culture supernatants, vesicles alone were able to reduce epithelial cell viability relative to untreated controls. Similar findings have been reported in previous studies describing vesicles released by toxigenic *C. difficile* strains, where vesicle preparations were shown to induce inflammatory responses and epithelial damage despite the absence of detectable toxin levels in vesicle proteomes (Nicholas et al., 2017; Caballano-Infantes et al., 2023; Fan et al., 2026). These observations support the concept that bacterial vesicles can function as independent biological particles capable of modulating host responses. The PNPG-based enzymatic assay performed in this study further revealed measurable glucosyltransferase activity associated with vesicle preparations produced under high salinity conditions. Although purified toxins TcdA and TcdB displayed substantially higher enzymatic activity, NaCl-derived vesicles generated detectable PNPG hydrolysis that increased following vesicle disruption by sonication. This observation suggests that enzymatically active components may be partially enclosed within vesicles and become more accessible following vesicle disruption. Although the increase in activity after vesicle disruption is consistent with protected intravesicular cargo, we cannot exclude the contribution of vesicle-associated or co-purified proteins. Vesicle-mediated packaging of toxins or enzymatic proteins has been reported in several bacterial systems and may provide a mechanism for protecting virulence factors from extracellular degradation while facilitating their delivery to host cells (Rivera et al., 2010; Toyofuku et al., 2023). An additional limitation of the present study is that CMV preparations were obtained using differential ultracentrifugation without subsequent purification by density gradient separation or size exclusion chromatography. Therefore, it is possible that extracellular proteins present in the culture supernatant were co-purified with CMVs and contributed to the protein profiles observed under both experimental conditions. Consequently, the altered vesicle-associated protein patterns detected under osmotic stress remain speculative, as they may reflect a combination of vesicle-associated components and co-isolated extracellular proteins. Future studies incorporating higher-resolution purification strategies and targeted protein analyses will be required to definitively characterize CMV cargo composition under osmotic stress conditions. Although the present study demonstrates that osmotic stress alters CMV-associated protein profiles and epithelial cell interactions, the specific vesicular components responsible for these effects remain to be determined. In particular, characterization of toxin-associated proteins and surface-layer components using targeted immunodetection approaches would help clarify whether osmotic stress influences the selective enrichment of biologically relevant proteins within CMVs. Future studies combining higher-resolution vesicle purification strategies with antibody-based characterization and proteomic analyses will be important to further define the molecular composition and functional properties of stress-associated CMVs.

Taken together, the results of this study indicate that osmotic stress can influence the physicochemical characteristics and host interaction properties of vesicles produced by *C. difficile*. Growth under high-salinity conditions was associated with smaller vesicle diameters, altered protein and lipid composition, increased epithelial cell association and detectable toxin-associated enzymatic activity. Although the precise mechanisms underlying these changes remain to be determined, environmental stress conditions such as osmotic pressure may influence vesicle biogenesis and cargo selection through effects on membrane structure and bacterial stress response pathways.

Future studies should therefore focus on identifying the molecular cargo of vesicles produced under different environmental conditions using proteomic and lipidomic approaches. The differential protein banding patterns observed by SDS–PAGE suggest that osmotic stress is associated with changes in CMV-associated protein composition. Although electrophoretic profiles do not allow direct protein identification, we could speculate the presence of proteins commonly reported in bacterial vesicles, including S-layer and cell-wall-associated proteins, stress-related chaperones such as GroEL and DnaK, and abundant metabolic enzymes with potential moonlighting functions (Brown et al., 2015; Toyofuku et al., 2019; Fagan and Fairweather, 2014; Bradshaw et al., 2017; Rivera et al., 2010; Henderson and Martin, 2013; Jeffery, 2018). These protein classes have been implicated in host–cell interaction and may contribute to the increased epithelial association observed under high-salinity conditions. Notably, while high-molecular-weight toxins such as TcdA and TcdB represent key virulence factors in *C. difficile*, their presence cannot be inferred from SDS–PAGE alone (Aktories et al., 2017; Kordus et al., 2022), and targeted molecular approaches would be required to assess their association with CMVs (Rasti and Brown, 2019). Therefore, the observed differences in protein profiles likely reflect broader stress-associated remodeling of vesicle cargo rather than the selective enrichment of specific virulence determinants. Determining whether toxins such as TcdA or TcdB are selectively packaged into vesicles under specific environmental conditions will be particularly important for understanding the potential role of vesicles in toxin delivery during infection. Additionally, investigation of vesicle-mediated host interactions in more physiologically relevant models, including organoid systems or *in vivo* infection models, will provide further insight into the contribution of vesicles to *C. difficile* pathogenesis.

In conclusion, our findings indicate that osmotic stress influences the production and functional properties of cytoplasmic membrane vesicles released by *C. difficile* R20291. High-salinity conditions were associated with changes in vesicle size distribution, biochemical composition and epithelial cell association, supporting a role for environmental factors in modulating vesicle-mediated processes during bacterial adaptation to host environments. In addition, our results are consistent with the presence of membrane-protected enzymatic activity associated with CMV preparations. Together, these findings support a model in which osmotic stress remodels CMV physicochemical properties and contributes to their functional interaction with host cells.

## Data Availability

The original contributions presented in this study are included in this article/Supplementary material, further inquiries can be directed to the corresponding authors.

## Supplementary material

Supplementary Figure 1 Confocal fluorescence microscopy controls for background fluorescence and dye autofluorescence. Representative confocal micrographs of epithelial cells stained to evaluate background fluorescence and potential DiO autofluorescence in the absence of cytoplasmic membrane vesicles (CMVs). Cell nuclei were stained with Hoechst 33342 (blue) and the plasma membrane was labeled with wheat germ agglutinin (WGA) conjugated to a red fluorophore. **(a–c)** Autofluorescence control for the DiO channel. Cells stained with Hoechst **(a)** and WGA **(b)** were imaged in the DiO detection channel **(c)** to assess potential background or autofluorescent signal in the green channel under the same acquisition settings used for vesicle experiments. **(d–f)** Negative cellular control without CMVs. Cells stained with Hoechst **(d)** and WGA **(e)** were imaged in the DiO channel **(f)** in the absence of DiO-labeled vesicles, confirming the lack of detectable fluorescence signal in the green channel under experimental conditions. Images were acquired using identical confocal microscopy settings across all panels. Scale bars represent 10 μm.

Supplementary Figure 2 Flow cytometry gating strategy and quantification of DIO-positive cells following exposure to bacterial vesicles without Trypan blue quenching. **(A)** Sequential gating strategy used for all samples. Cells were first identified based on forward scatter (FSC) and side scatter (SSC) to exclude debris. Doublets were excluded by gating on FSC-area versus FSC-height to select single cells. Live cells were identified using viability dye exclusion. Representative dot plot showing discrimination of DIO-negative (blue) and DIO-positive (red) populations. Importantly, only viable cells were included in the final analysis of DIO-associated fluorescence. **(B)** Representative density plots illustrating DIO fluorescence intensity in HT-29 cells under different experimental conditions. The percentage of DIO-positive (DIO^+^) cells is indicated in each panel. The vertical gate defines the threshold for DIO positivity, established using untreated HT-29 cells and unstained negative controls. Across conditions, a progressive increase in DIO^+^ cells were observed, indicating increased CMV-associated fluorescence with epithelial cells. Untreated HT-29 cells showed minimal background fluorescence (<1% DIO^+^), confirming the specificity of the staining and gating strategy.
