# Supplementary material for: Cytoplasmic membrane vesicles from Clostridioides difficile R20291 are remodeled by osmotic stress
Source: Front Microbiol. 2026 Jun 17;17:1868783. doi: 10.3389/fmicb.2026.1868783 (PMC13318942; doi:10.3389/fmicb.2026.1868783)
Supplement: Supplementary file 1 [file Table_1.docx]

**Table S1: Nanoparticle tracking analysis (NTA) parameters describing size distribution and concentration of cytoplasmic membrane vesicles (CMVs) produced by *Clostridioides difficile* R20291 under control (CTRL) and high-salinity (NaCl) conditions.**

| **Parameter** | **CTRL** | **NaCl (350 mM)** | **Interpretation** |
| --- | --- | --- | --- |
| **Mean size (nm)** | 145.6 ± 1.2 | 108.7 ± 3.4 | NaCl CMVs exhibit smaller size |
| **Mode (nm)** | 92.6 | 84.7 | Shift toward smaller CMVs in NaCl |
| **SD (nm)** | 56.3 | 40.6 | NaCl CMVs are less polydisperse |
| **D10 (nm)** | 90.1 | 75.1 | Increased proportion of smaller vesicles in NaCl |
| **D50 (nm)** | 130.8 | 100.1 | Median size reduced in NaCl-derived CMVs |
| **D90 (nm)** | 222.8 | 166.6 | Reduced abundance of larger vesicles in NaCl |
| **Concentration (particles/mL)** | 7.60 × 10¹¹ | 4.39 × 10¹¹ | CTRL has ~1.7× higher yield |
| **Particles/frame** | 41.5 | 48.0 | Both within optimal detection range |
| **Centers/frame** | 47.7 | 52.0 | Both within optimal detection range |
