# Supplementary figures and images for: Cytoplasmic membrane vesicles from Clostridioides difficile R20291 are remodeled by osmotic stress
Source: Front Microbiol. 2026 Jun 17;17:1868783. doi: 10.3389/fmicb.2026.1868783 (PMC13318942; doi:10.3389/fmicb.2026.1868783)

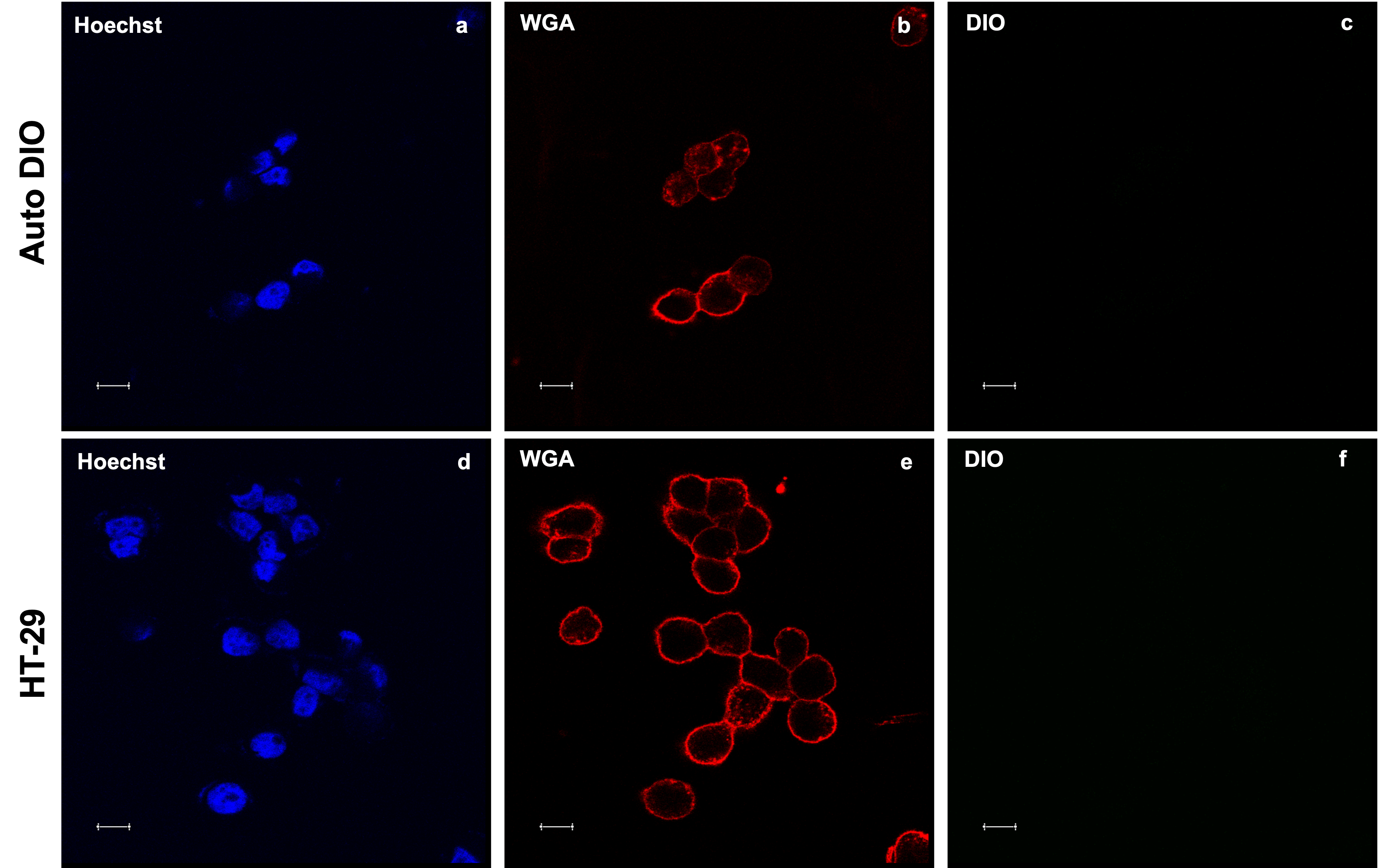

Supplement: Supplementary Figure 1 — Confocal fluorescence microscopy controls for background fluorescence and dye autofluorescence. Representative confocal micrographs of epithelial cells stained to evaluate background fluorescence and potential DiO autofluorescence in the absence of cytoplasmic membrane vesicles (CMVs). Cell nuclei were stained with Hoechst 33342 (blue) and the plasma membrane was labeled with wheat germ agglutinin (WGA) conjugated to a red fluorophore. (a–c) Autofluorescence control for the DiO channel. Cells stained with Hoechst (a) and WGA (b) were imaged in the DiO detection channel (c) to assess potential background or autofluorescent signal in the green channel under the same acquisition settings used for vesicle experiments. (d–f) Negative cellular control without CMVs. Cells stained with Hoechst (d) and WGA (e) were imaged in the DiO channel (f) in the absence of DiO-labeled vesicles, confirming the lack of detectable fluorescence signal in the green channel under experimental conditions. Images were acquired using identical confocal microscopy settings across all panels. Scale bars represent 10 μm. [file Image_1.tiff]

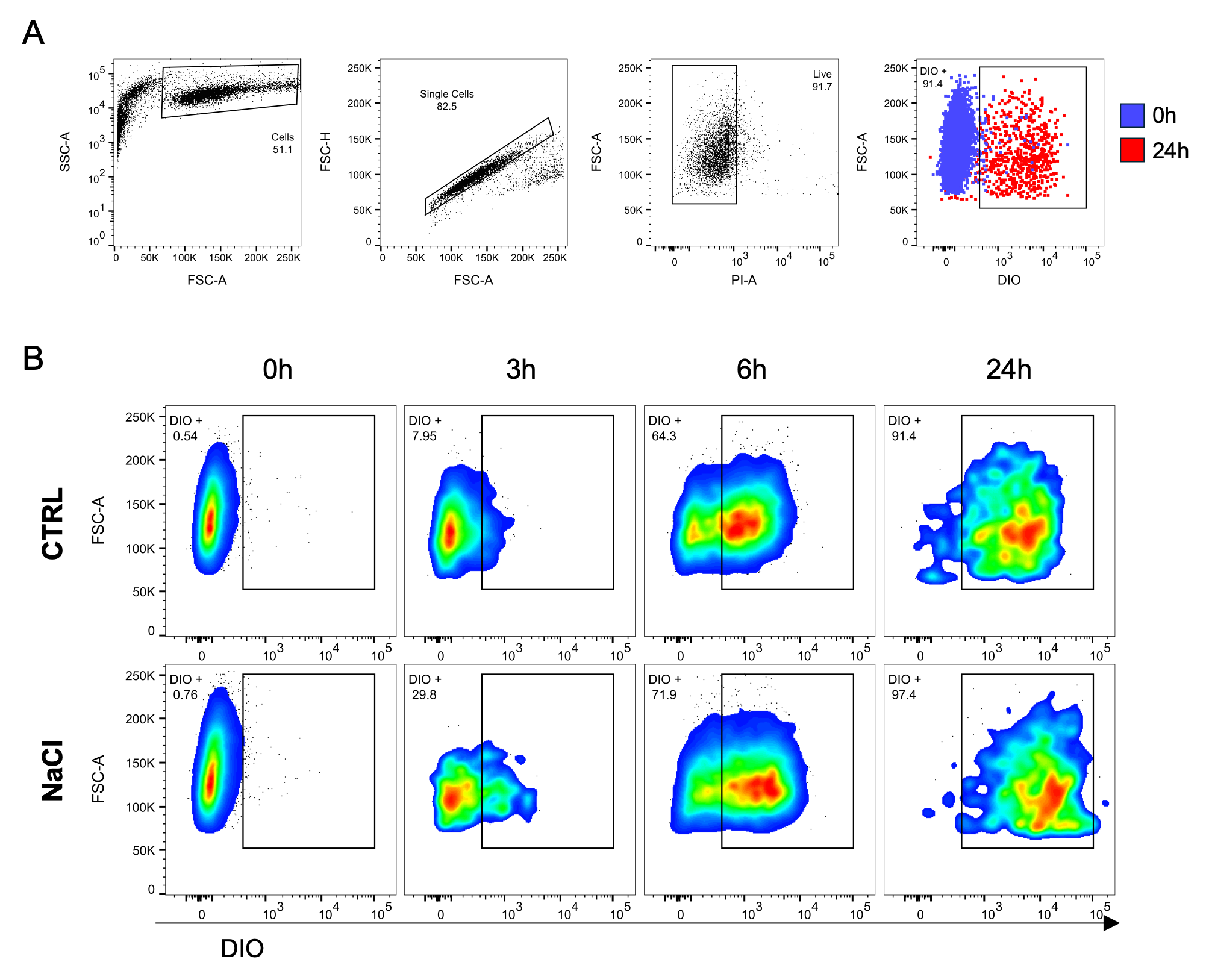

Supplement: Supplementary Figure 2 — Flow cytometry gating strategy and quantification of DIO-positive cells following exposure to bacterial vesicles without Trypan blue quenching. (A) Sequential gating strategy used for all samples. Cells were first identified based on forward scatter (FSC) and side scatter (SSC) to exclude debris. Doublets were excluded by gating on FSC-area versus FSC-height to select single cells. Live cells were identified using viability dye exclusion. Representative dot plot showing discrimination of DIO-negative (blue) and DIO-positive (red) populations. Importantly, only viable cells were included in the final analysis of DIO-associated fluorescence. (B) Representative density plots illustrating DIO fluorescence intensity in HT-29 cells under different experimental conditions. The percentage of DIO-positive (DIO+) cells is indicated in each panel. The vertical gate defines the threshold for DIO positivity, established using untreated HT-29 cells and unstained negative controls. Across conditions, a progressive increase in DIO+ cells were observed, indicating increased CMV-associated fluorescence with epithelial cells. Untreated HT-29 cells showed minimal background fluorescence (<1% DIO+), confirming the specificity of the staining and gating strategy. [file Image_2.tiff]
